# Supplementary material for: Associations Between Carbohydrate Intake Behaviours and Glycaemia in Gestational Diabetes: A Prospective Observational Study
Source: Nutrients. 2025 Jan 22;17(3):400. doi: 10.3390/nu17030400 (PMC11819901; doi:10.3390/nu17030400)
Supplement: Supplementary file 1 [file nutrients-17-00400-s001.zip › Supplementary Materials Table S1-S5.pdf]

Supplementary Tables

Manuscript Title: Associations Between Carbohydrate Intake Behaviours and Glycaemia in Gestational Diabetes: A Prospective Observational Study

Supplementary Table S1. Additional dietary, blood glucose and other descriptives for individuals with gestational diabetes the week following initial dietary group education [n=97; median (IQR) or n (%)]

Baseline characteristics:		Carbohydrate intake behaviours:	>2 episodes per week
Pre-pregnancy body mass index		Carbohydrate <175g /day	73 (75.26%)
- <18.0 (kg/m^2)	2 (2.11%)	Overnight fast (>10 hr)	89 (94.98%)
- 18.0-24.9 (kg/m^2)	43 (45.26%)	Daytime fast (>3.5 hours)	87 (89.69%)
- 25.0-29.9 (kg/m^2)	29 (30.53%)	- Morning	53 (54.64%)
- ≥30.0 (kg/m^2)	21 (22.11%)	- Afternoon	63 (64.95%)
Nulliparous	37 (38.14%)	- Evening	19 (19.59%)
Previous gestational diabetes	16 (16.49%)		
Ethnicity self reported		Time variability (SD[a] >1.5 hr):	
- European	6 (6.19%)	- Out of bed	11 (11.34%)
- Pacific islands	6 (6.19%)	- Into bed	9 (9.78%)
- Australian aboriginal	1 (1.03%)		
- African	1 (1.03%)	Computerised analysis:	
- South American	1 (1.03%)5.1	Energy (kJ/day)	8206 (6707-10093)
Haemoglobin A1c (%)	(4.9-5.3)	Carbohydrate (g/day)	210 (174-250)
		Protein (g/day)	96 (77-118)
Blood glucose levels:		Fat (g/day)	73 (54-95)
Variability (SD[a] >0.5 mmol/L)		Dietary fibre (g/day)	24 (19-32)
- Fasting	3 (3.13%)		
- Post Breakfast	66 (68.75%)	Weight change in the week:	
- Post Lunch	76 (79.17%)	> 0.5 kg loss	12 (16.00%)
- Post Dinner	74 (78.72%)	> 0.5 kg gain	14 (18.67%)
Procedure errors (>2 per week):			
- Any	60 (61.86%)		
- Missed test	15 (15.46%)		
- Late fasting test[b]	14 (14.43%)		
- Late PP test (>130 min)	32 (32.99%)		
- Early PP test (<110 min)	1 (1.03%)		
- Additional eating prior to test	5 (5.15%)		
Incorrectly reported	3 (3.09%)		

[a] SD for the individual; [b] Out of bed more than 0.5 hour before BG test

Supplementary Table S2. Non-significant dietary and other relevant associations for individuals with clinically elevated verses in-target fasting glycaemia (p≥0.05)

**Variables**	**Clinically elevated[a] fasting glycaemia** n=48, median (IQR)	**Clinically in-target fasting glycaemia** n=49, median (IQR)	***p* value**
Maternal age (years)	34 (29-37)	31 (28-34)	0.066
Pre-pregnancy body mass index (kg/m^2)	26.1 (23.4-30.2)	24.6 (22.3-28.1)	0.172
Previous gestational diabetes	7 (14.58%)	9 (18.37%)	0.616
Nulliparity	16 (33.33%)	21 (42.86%)	0.334
Self reported ethnicity	-	-	0.666
- South Asia	14 (29.17%)	15 (30.61%)	-
- South East Asia	7 (14.58%)	13 (26.53%)	-
- Middle East	10 (20.83%)	9 (18.37%)	-
- Anglo-Australian	8 (16.67%)	6 (12.24%)	-

Variables	Clinically elevated[a] fasting glycaemia n=48, median (IQR)	Clinically in-target fasting glycaemia n=49, median (IQR)	*p* value
- Combined Asian[b] vs non Asian	21 (43.75%)	28 (57.14%)	0.187
Gestational age (weeks)	24 (17-30)	28 (22-29)	0.112
OGTT[c] 1 hour level (mmol/L)	10.0 (8.3-11.4)	9.9 (8.3-10.6)	0.420
OGTT 2 hour level (mmol/L)	7.4 (6.1-9.0)	7.9 (6.9-8.8)	0.488
Postprandial BG[d] elevations (per 7 days)	5.75 (2.74 – 10.50)	3.15 (0.00-8.23)	0.070
BG procedure errors (per 7 days)	4.38 (1.75-8.66)	3.88 (1.65-6.22)	0.611
Weight change in the study week (kg)	0.10 (-0.30 to 0.43)	0.05 (-0.33 to 0.29)	0.417
**Manual dietary analysis:**			
Number of eating episodes (per day)	5.38 (4.69-5.77)	5.50 (4.88-6.13)	0.194
Carbohydrate serves[e] per lunch	2.79 (2.35-3.29)	2.93 (2.43-3.69)	0.285
Carbohydrate serves per snack	1.23 (1.10-1.51)	1.38 (1.10-1.57)	0.571
Missed breakfasts (per 7 days)	0.00 (0.00-0.78)	0.00 (0.00-0.78)	0.868
Low carbohydrate			
- breakfasts (<30g per 7 days)	0.88 (0.00-2.33)	0.88 (0.00-1.75)	0.695
- lunches (<30g per 7 days)	1.00 (0.00-1.81)	0.78 (0.00-1.75)	0.110
- snacks (<15g per 7 days)	2.48 (0.88-3.56)	2.00 (0.88-4.00)	0.882
High carbohydrate			
- lunches (>50g per 7 days)	1.75 (0.88-3.03)	2.00 (0.88-3.00)	0.572
- snacks (>50g per 7 days)	1.75 (0.00-2.81)	2.00 (0.88-3.40)	0.283
High glycaemic index meals (per 7 days)	2.63 (1.46-4.85)	3.50 (1.00-6.13)	0.699
Variability in timing of meals (SD[f], hr)	1.00 (0.79-1.39)	0.97 (0.69-1.27)	0.229
Overnight fasting time (hr)	10.92 (10.00-11.82)	10.71 (9.68-11.91)	0.564
Day fasts >3.5 hours (per 7 days)	6.50 (4.25-10.43)	7.88 (5.15-10.00)	0.473
**Computerised (3 day) analysis:**			
Energy (kJ per day)	8063 (6584-10250)	8342 (7005-9894)	0.534
Fat (g per day)	77 (57-102)	72 (54-93)	0.559
Protein (g per day)	97 (77-119)	96 (79-115)	0.715
- Energy from protein (%)	19.95 (18.58-22.96)	19.64 (17.10-22.03)	0.129
Dietary fibre (g per day)	23 (19-31)	26 (19-32)	0.605
Food group (serves[g] per day)			
- vegetables	3.35 (1.83-4.47)	2.84 (1.97-5.25)	0.862
- fruit	0.87 (0.43-1.59)	1.33 (0.76-1.77)	0.070
- dairy	1.50 (1.03-2.08)	1.53 (0.94-2.24)	0.607
- protein	2.99 (1.97-3.99)	2.47 (1.98-3.13)	0.324
Micronutrients (per day)			
- Total vitamin A equivalents (mg)	676 (506-984)	909 (466-1437)	0.171
- Thiamin (mg)	1.34 (1.01-1.79)	1.30 (1.00-1.83)	0.985
- Riboflavin (mg)	1.68 (1.25-2.12)	1.66 (1.26-2.31)	0.922
- Niacin equivalents (mg)	41.57 (34.35-54.90)	39.57 (32.19-47.40)	0.241
- Potassium (mg)	2840 (2239-3510)	2782 (2252-3573)	0.955
- Vitamin C (mg)	73.63 (40.63-114.50)	74.27 (47.16-121.16)	0.585
- Magnesium (mg)	305 (224-380)	306 (244-370)	0.854
- Calcium (mg)	749 (539-948)	752 (571-992)	0.565
- Phosphorus (mg)	1409 (1218-1790)	1449 (1227-1694)	0.883
- Iron (mg)	10.04 (7.91-13.87)	10.22 (7.93-13.17)	0.844
- Zinc (mg)	10.73 (8.60-12.80)	11.16 (8.95-13.41)	0.783
- Vitamin E (mg)	10.61 (7.99-16.58)	11.58 (8.51-15.44)	0.755
- Vitamin B12 (μg)	3.85 (3.13-5.15)	4.51 (3.14-5.91)	0.313
- Total folate (μg)	492 (397-616)	482 (426-607)	0.709
- Selenium (μg)	88 (67-109)	88 (65-105)	0.738
- Vitamin B6 (μg)	1.86 (1.07-2.29)	1.81 (1.48-2.42)	0.302
**Non-dietary episodes** (per 7 days):			
Poor sleep	0.00 (0.00-0.91)	0.00 (0.00-0.88)	0.784
Hunger	0.00 (0.00-0.88)	0.00 (0.00-0.88)	0.993

Variables	Clinically elevated[a] fasting glycaemia n=48, median (IQR)	Clinically in-target fasting glycaemia n=49, median (IQR)	*p* value
Stress	0.00 (0.00-0.22)	0.00 (0.00-1.00)	0.275
Physical activity	2.19 (0.00-5.28)	1.75 (0.00-4.86)	0.851

[a] More than 20% of readings >5.0 mmol/L. [b] South Asian and South East Asian. [c] 75g oral glucose tolerance test. [d] Blood glucose. [e]One serve = 15g carbohydrate. [f] SD for the individual. [g] Serve sizes as per FoodWorks (Version 9, Xyris Software). *p<0.05, **p<0.01 ***p<0.001

Supplementary Table S3. Non-significant dietary and other relevant associations for individuals with clinically elevated verses in-target postprandial glycaemia ($p \geq 0.05$)

Variables[a]	Clinically elevated[b] postprandial glycaemia n=52	Clinically in-target postprandial glycaemia n=45	*p* value
Maternal age (years)	33 (30 -37)	31 (27-34)	0.127
Pre-pregnancy			
- weight (kg)	64.0 (57.8 -73.3)	64.0 (59.5-78.8)	0.601
- body mass index (kg/m^2)	25.1 (22.7 -28.4)	25.3 (22.9-30.1)	0.858
OGTT[c] fasting glucose level (mmol/L)	4.8 (4.6 -5.3)	5.1 (4.7-5.2)	0.375
Haemoglobin A1c (%)	5.2 (4.9-5.4)	5.0 (4.9-5.1)	0.081[d]
Gestational age (weeks)	27.5 (18.0-30.0)	28.0 (20.00-30.00)	0.518
Previous gestational diabetes	10 (19.23%)	6 (13.33%)	0.435
Nulliparity	32 (61.54%)	28 (62.22%)	0.945
Self reported ethnicity	-	-	0.768
- South Asia	17 (32.69%)	12 (26.67%)	-
- South East Asia	12 (23.08%)	8 (17.78%)	-
- Middle East	9 (17.31%)	10 (22.22%)	-
- Anglo-Australian	7 (13.46%)	7 (15.56%)	-
- Combined Asian[e] vs non Asian	29 (55.77%)	20 (44.44%)	0.266
Fasting BG elevations (per 7 days)	1.75 (0.66 -5.06)	1.00 (0.00-4.00)	0.102
BG procedure errors (per 7 days)	4.44 (1.00 -8.64)	3.50 (2.00-6.63)	0.925
Current weight (kg)	69.4 (62.2 -80.7)	73.7 (65.5-86.4)	0.289
Weight change in the study week (kg)	0.10 (-0.14 to 0.51)	0.00 (-0.35 to 0.30)	0.133
**Manual dietary analysis:**			
Eating episodes per day	5.33 (4.63 -6.00)	5.50 (4.88-6.13)	0.161
Carb. serves[f] per day			
- <9 (number per 7 days)	0.94 (0.00 -2.85)	0.88 (0.00-2.33)	0.413
- >12 (number per 7 days)	3.00 (0.88 -4.75)	3.00 (0.88-4.67)	0.959
Carb. serves per meal			
Carb. serves per snack	1.26 (1.09 -1.50)	1.32 (1.10-1.57)	0.778
Missed meals (per 7 days)	1.00 (0.00-4.38)	0.78 (0.00-1.17)	0.078
Missed snacks (per 7 days)	9.31 (6.20 -14.88)	7.88 (5.60-12.25)	0.159
Low carb. (per 7 days)			
- meals (<30g)	3.03 (1.46 -6.27)	2.63 (1.75-5.25)	0.811
- snacks (<15g)	1.94 (0.88 -4.00)	2.63 (0.88-3.50)	0.994
High carb. (>50g) meals (per 7 days)	4.67 (2.91 -7.88)	4.75 (2.53-7.88)	0.948
Variability in timing of meals (SD[g], hr)	0.95 (0.75 -1.38)	1.02 (0.80-1.28)	0.705
Variability in carb. at meals (SD[g], serves)	1.34 (1.09 -1.69)	1.18 (1.02-1.54)	0.189
Overnight fasting time (hr)	11.89 (10.42 -12.98)	11.93 (11.01-13.24)	0.467
Day fasts >3.5 hours (per 7 days)	7.00 (4.81 -11.38)	7.88 (4.38-10.00)	0.366
**Computerised (3 day) analysis:**			
Energy per kg (kJ per day)	106 (84.13-133.46)	119 (90-144)	0.199
Protein per kg (g per day)	1.27 (1.03-1.53)	1.38 (1.02-1.75)	0.199

Variables[a]	Clinically elevated[b] postprandial glycaemia n=52	Clinically in-target postprandial glycaemia n=45	*p* value
- Energy from protein (%)	19.95 (17.98-22.73)	19.65 (17.81-22.03)	0.820
Dietary fibre (g per day)	23.40 (18.60-32.02)	25.82 (21.20-33.65)	0.155
Micronutrients (per day)			
- Thiamin (mg)	1.22 (0.93-1.64)	1.54 (1.02-1.86)	0.186
- Riboflavin (mg)	1.57 (1.15-2.08)	1.74 (1.36-2.37)	0.106
- Vitamin C (mg)	66.84 (41.43-114.91)	78.95 (47.05-118.77)	0.386
- Iodine (μg)	152 (116-196)	177 (142-205)	0.096
- Vitamin B6 (mg)	1.63 (1.28-2.27)	2.09 (1.50-2.49)	0.147
Food group (serves[h] per day)			
- fruit	1.09 (0.54-1.63)	1.16 (0.61-1.79)	0.543
- grains	7.79 (5.01-10.04)	7.19 (5.60-8.83)	0.655
- vegetables	3.03 (1.70-4.40)	3.05 (2.19-5.09)	0.388
- dairy	1.30 (0.90-1.97)	1.93 (1.05-2.37)	0.076
- protein	2.37 (1.92-3.23)	2.85 (2.06-3.78)	0.135
**Non-dietary episodes** (per 7 days):			
Poor sleep	0.00 (0.00-0.88)	0.00 (0.00-1.00)	0.920
Hunger	0.00 (0.00-0.91)	0.00 (0.00-0.88)	0.835
Stress	0.00 (0.00-0.88)	0.00 (0.00-0.78)	0.688
Physical activity	0.88 (0.00-5.25)	3.00 (0.00-5.00)	0.375

All data presented as Median (IQR) or n (%). [a] All meals combined unless otherwise stated. [b] More than 20% of readings > 6.7mmol/L. [c] 75g oral glucose tolerance test. [d] Parametric data (ShapiroWilk test $p= 0.364$): Mean 5.2% (SD 0.34) vs 5.0% (SD 0.28), $p=0.052$ using Student T-test. [e]South Asian and South East Asian. [f] Carbohydrate serves; one serve = 15g. [g] SD for the individual. [h] Serve sizes as per FoodWorks (Version 9, Xyris Software). $^{*}p<0.05$, $^{**}p<0.01$ $^{***}p<0.001$

Supplementary Table S4. Summary of associations between elevated glycaemia and oral glucose tolerance test (OGTT) results, haemoglobin A1c, pre-pregnancy weight and body mass index (BMI).

	Elevated fasting glycaemia[a]	Elevated postprandial glycaemia[a]
OGTT[b] 0 hr	Significant (*p*=0.004)	Non significant (*p*=0.375)
OGTT 1 hr	Non significant (*p*=0.420)	Significant (*p*=0.01)
OGTT 2 hr	Non significant (*p*=0.488)	Significant (*p*<0.001)
HbA1c	Significant (*p*=0.003)	Borderline non-significant (*p*=0.05)
Pre-pregnancy weight	Significant (*p*=0.032)	Non significant (*p*=0.601)
Pre-pregnancy BMI	Non significant (*p*=0.172)	Non significant (*p*=0.858)

[a]>20% SMBG readings elevated within first week of gestational diabetes management. [b] 75g Oral glucose tolerance test

Supplementary Table S5: STrengthening the Reporting of OBservational studies in Epidemiology – Nutritional Epidemiology (STROBE-nut) Checklist

Item	Item nr	STROBE recommendations	Extension for Nutritional Epidemiology studies (STROBE-nut)	Reported on page #
**Title and abstract**	1	(a) Indicate the study's design with a commonly used term	**nut-1** State the dietary/nutritional assessment method(s)	**1**

Item		Item nr	STROBE recommendations	Extension for Nutritional Epidemiology studies (STROBE-nut)	Reported on page #
			in the title or the abstract.<br>(b) Provide in the abstract an informative and balanced summary of what was done and what was found.	used in the title, abstract, or keywords.	
**Introduction**					**2-4**
	Background rationale	2	Explain the scientific background and rationale for the investigation being reported.		**2-4**
	Objectives	3	State specific objectives, including any pre-specified hypotheses.		**4**
**Methods**					**4-6**
	Study design	4	Present key elements of study design early in the paper.		**1,4**
	Settings	5	Describe the setting, locations, and relevant dates, including periods of recruitment, exposure, follow-up, and data collection.	**nut-5** Describe any characteristics of the study settings that might affect the dietary intake or nutritional status of the participants, if applicable.	**4 Section 2.1**
	Participants	6	a) Cross-sectional study—Give the eligibility criteria, and the sources and methods of selection of participants.	**nut-6** Report particular dietary, physiological or nutritional characteristics that were considered when selecting the target population.	**4, 5**<br>**Section 2.2-2.3**
	Variables	7	Clearly define all outcomes, exposures, predictors, potential confounders, and effect modifiers. Give diagnostic criteria, if applicable.	**nut-7.1** Clearly define foods, food groups, nutrients, or other food components.<br>**nut-7.2** When using dietary patterns or indices, describe the methods to obtain	**5,6**<br>**Section 2.4, 2.5**

Item		Item nr	STROBE recommendations	Extension for Nutritional Epidemiology studies (STROBE-nut)	Reported on page #
				them and their nutritional properties.	
	Data sources - measurements	8	For each variable of interest, give sources of data and details of methods of assessment (measurement). Describe comparability of assessment methods if there is more than one group.	**nut-8.1** Describe the dietary assessment method(s), e.g., portion size estimation, number of days and items recorded, how it was developed and administered, and how quality was assured. Report if and how supplement intake was assessed.<br>**nut-8.2** Describe and justify food composition data used. Explain the procedure to match food composition with consumption data. Describe the use of conversion factors, if applicable.<br>**nut-8.5** Describe the assessment of nondietary data (e.g., nutritional status and influencing factors) and timing of the assessment of these variables in relation to dietary assessment.<br>**nut-8.6** Report on the validity of the dietary or nutritional assessment methods and any internal or external validation used in the study, if applicable.	**5 Section 2.4**
	Bias	9	Describe any efforts to address potential sources of bias.	**nut-9** Report how bias in dietary or nutritional assessment was addressed, e.g., misreporting, changes	**-**

Item		Item nr	STROBE recommendations	Extension for Nutritional Epidemiology studies (STROBE-nut)	Reported on page #
				in habits as a result of being measured, or data imputation from other sources	
	Study Size	10	Explain how the study size was arrived at.		**4**<br>**Section 2.2**
	Quantitative variables	11	Explain how quantitative variables were handled in the analyses. If applicable, describe which groupings were chosen and why.	**nut-11** Explain categorization of dietary/nutritional data (e.g., use of N-tiles and handling of nonconsumers) and the choice of reference category, if applicable.	**5,6**<br>**Section 2.4, 2.6**
	Statistical Methods	12	(a) Describe all statistical methods, including those used to control for confounding<br>(b) Describe any methods used to examine subgroups and interactions.<br>(c) Explain how missing data were addressed.<br>(d) Cross-sectional study—If applicable, describe analytical methods taking account of sampling strategy.<br>(e) Describe any sensitivity analyses.	**nut-12.1** Describe any statistical method used to combine dietary or nutritional data, if applicable.<br>**nut-12.2** Describe and justify the method for energy adjustments, intake modeling, and use of weighting factors, if applicable.<br>**nut-12.3** Report any adjustments for measurement error, i.e,. from a validity or calibration study.	**6**<br>**Section 2.6**
**Results**					**6-12**
	Participants	13	(a) Report the numbers of individuals at each stage of the study—e.g., numbers potentially eligible, examined for eligibility, confirmed eligible, included in the study, completing follow-up, and	**nut-13** Report the number of individuals excluded based on missing, incomplete or implausible dietary/nutritional data.	**6**<br>**Section 3.1**

Item		Item nr	STROBE recommendations	Extension for Nutritional Epidemiology studies (STROBE-nut)	Reported on page #
			analyzed.<br>(b) Give reasons for non-participation at each stage.<br>(c) Consider use of a flow diagram.		
	Descriptive data	14	(a) Give characteristics of study participants (e.g., demographic, clinical, social) and information on exposures and potential confounders<br>(b) Indicate the number of participants with missing data for each variable of interest	**nut-14** Give the distribution of participant characteristics across the exposure variables if applicable. Specify if food consumption of total population or consumers only were used to obtain results.	**7,8**<br>**Table 1**<br>**Supplementary Table 1**
	Outcome data	15	Cross-sectional study—Report numbers of outcome events or summary measures.		**8-12,**<br>**Sections 3.2 – 3.5**<br>**Tables 2-5**<br>**Supplementary tables 2-4**
	Main results	16	(a) Give unadjusted estimates and, if applicable, confounder-adjusted estimates and their precision (e.g., 95% confidence interval). Make clear which confounders were adjusted for and why they were included.<br>(b) Report category boundaries when continuous variables were categorized.<br>(c) If relevant, consider translating estimates of relative risk into absolute risk for a meaningful time period.	**nut-16** Specify if nutrient intakes are reported with or without inclusion of dietary supplement intake, if applicable.	**8-12, Sections 3.2 – 3.5**<br>**Tables 2-5**
	Other analyses	17	Report other analyses	**nut-17** Report any	**-**

Item		Item nr	STROBE recommendations	Extension for Nutritional Epidemiology studies (STROBE-nut)	Reported on page #
			done—e.g., analyses of subgroups and interactions and sensitivity analyses.	sensitivity analysis (e.g., exclusion of misreporters or outliers) and data imputation, if applicable.	
**Discussion**					**12-18**
	Key results	18	Summarize key results with reference to study objectives.		**12**
	Limitation	19	Discuss limitations of the study, taking into account sources of potential bias or imprecision. Discuss both direction and magnitude of any potential bias.	**nut-19** Describe the main limitations of the data sources and assessment methods used and implications for the interpretation of the findings.	**16**<br>**Section 4.8**
	Interpretation	20	Give a cautious overall interpretation of results considering objectives, limitations, multiplicity of analyses, results from similar studies, and other relevant evidence.	**nut-20** Report the nutritional relevance of the findings, given the complexity of diet or nutrition as an exposure.	**12-18**
	Generalizability	21	Discuss the generalizability (external validity) of the study results.		**16**<br>**Section 4.8**
**Other information**					
	Funding	22	Give the source of funding and the role of the funders for the present study and, if applicable, for the original study on which the present article is based.		**18**
	Ethics			**nut-22.1** Describe the procedure for consent and study approval from ethics committee(s).	**4,6, 18**<br>**Section 2.2, 2.7**
	Supplementary			**nut-22.2** Provide data	**18**

Item	Item nr	STROBE recommendations	Extension for Nutritional Epidemiology studies (STROBE-nut)	Reported on page #
material			collection tools and data as online material or explain how they can be accessed.	
