# Supplementary material for: Associations Between Carbohydrate Intake Behaviours and Glycaemia in Gestational Diabetes: A Prospective Observational Study
Source: Nutrients. 2025 Jan 22;17(3):400. doi: 10.3390/nu17030400 (PMC11819901; doi:10.3390/nu17030400)
Supplement: Supplementary file 1 [file nutrients-17-00400-s001.zip › Supplementary Materials Table S6. Food and SMBG Diary.pdf]

XXXX HOSPITAL GDM FOOD & GLUCOSE DIARY

This is completed by all English literate GDM patients the week following their GDM education seminar, as part of existing routine practice.

Gestational Diabetes

Food

and

Glucose Diary

What is the Purpose of this Diary?

Blood glucose is affected by many things. For example:

- **Food** (especially too much or not enough carbohydrate food)
- **Physical activity** (this usually lowers blood glucose)
- **Stress, illness, pain, poor sleep** (these often raise blood glucose)
- **Changing pregnancy hormones** (these may raise or lower blood glucose from day to day)

This diary is a tool to see how your body is responding to these factors. Every person is different. What raises blood glucose in one person, may not in another person. It is definitely not a test, to see if you have been “good” or “bad”. It is a good opportunity to learn more about your body.

The diary will help your Diabetes Health Workers give you the best advice – tailored just for you.

For How Long do I use the Diary?

We suggest you fill out the diary for one week (7-9 days). Bring the diary to your next appointment for your Diabetes Educator and Dietitian to review. After that, you will use a simpler glucose diary for the rest of your pregnancy. On the simpler diary you only record foods if a glucose reading is high.

Tips for Filling out the Diary

- Try to fill out the diary as you go through the day. Take it with you when you are out. Don't leave it until the end of the day, or you may forget important information.
- Don't just tell us what you think we want to hear, as this will not help your baby. Please include everything.
- Food types and amounts are important. You don't need to weigh or measure your foods, but try to estimate how much you are eating with your eyes. We suggest you compare your food to a cup (250ml). This may be similar to the size of your fist. Is the food amount ½ cup, 1 cup, 1 ½ cups or 2 cups?
- Buy your blood strips as soon as possible. Otherwise you will run out after a few days, and we may not have enough information when you come to your review appointment. You can call XXX to find out your NDSS number. If you give this number to your pharmacist, you will get the strips at a cheap price.

Example:

**Physical Activity** above or below normal	Describe	Start Time	End Time
	Cleaned fridge	10.10	10.45
	Afternoon sleep	1.30	2.15

**Food and Drink** (except water)	**Time**	**Food/ Drink**	**Amount**
Between getting up and breakfast	7.40 am	Coffee (1/2 cup milk, no sugar)	
Breakfast	8.15 – 8.30 am		
		Toast – Burgen bread	2 slices
		Margarine	2 teaspoons
		Banana	1 medium

Hunger, Physical Activity and Stress/Illness/Pain

Please record episodes whenever they occur.
If there are no episodes, you can leave blank.

Food Amounts

Please write amounts in cups where you can, especially for carbohydrate foods.
For example: ½ cup, 1 cup, 1 ½ cup, 2 cups
1 cup is the size of an average person's fist (250 ml)

What if I have questions about the diary?

If you have any questions about this diary, please call the Diabetes Centre on 8738 4577, and ask to speak with a Diabetes Educator or Dietitian.

Day 1

Day ____ Date ______ Time I got out of bed ______am

**Physical Activity** above or below normal eg walking cleaning shopping afternoon sleep lie down	Describe	Start Time	End Time

**Hunger Episodes** 1 = light (I can ignore it) 2 = moderate (I can't ignore it) 3 = high (I can't control it)	Start Time	End Time	Score

**Stress, Illness or Pain** (S, I, P) 1 = a little (I can ignore it) 2 = moderate (I can't ignore) 3 = high (distressing)	S, I or P?	Start Time	End Time	Score

**Food and Drink** (except water)	**Time**	**Food/ Drink**	**Amount**
Between getting up and breakfast			
**Breakfast**			
Between breakfast and lunch (snacks)			
**Lunch**			
Between lunch and dinner (snacks)			
**Dinner**			
Between dinner and bed-time (snacks)			
Overnight			

**Blood Glucose**
Waking up reading:
2 hours after breakfast:
2 hours after lunch:
2 hours after dinner:

Time I went to bed ______pm

My sleep was

☐ Good

☐ OK

☐ Poor
